# Androgen serum levels in male patients with adrenocortical carcinoma given mitotane therapy: A single center retrospective longitudinal study

**DOI:** 10.3389/fendo.2023.1128061

**Published:** 2023-04-05

**Authors:** Andrea Delbarba, Deborah Cosentini, Paolo Facondo, Marta Laganà, Letizia Chiara Pezzaioli, Valentina Cremaschi, Andrea Alberti, Salvatore Grisanti, Carlo Cappelli, Alberto Ferlin, Alfredo Berruti

**Affiliations:** ^1^ Department of Experimental Sciences, Unit of Endocrinology and Metabolism, University of Brescia, Azienda Socio Sanitaria Territoriale (ASST) Spedali Civili, Brescia, Italy; ^2^ Department of Medical and Surgical Specialties, Radiological Sciences, and Public Health, Medical Oncology Unit, University of Brescia, ASST Spedali Civili, Brescia, Italy; ^3^ Unit of Andrology and Reproductive Medicine, Department of Medicine, University of Padua, Padua, Italy

**Keywords:** adrenal tumor, mitotane, androgens, hypogonadism, testosterone

## Abstract

**Objective:**

Hypogonadism is common in male patients with adrenocortical carcinoma (ACC) who are under treatment with mitotane, but the phenomenon is underestimated, and its prevalence has been poorly studied. This single-center retrospective longitudinal study was undertaken to assess the frequency of testosterone deficiency before and after mitotane therapy, the possible mechanism involved, and the relationship between hypogonadism with serum mitotane levels and prognosis.

**Research design and methods:**

Consecutive male ACC patients followed at the Medical Oncology of Spedali Civili Hospital in Brescia underwent hormonal assessment to detect testosterone deficiency at baseline and during mitotane therapy.

**Results:**

A total of 24 patients entered the study. Of these patients, 10 (41.7%) already had testosterone deficiency at baseline. During follow-up, total testosterone (TT) showed a biphasic evolution over time with an increase in the first 6 months followed by a subsequent progressive decrease until 36 months. Sex hormone binding globulin (SHBG) progressively increased, and calculated free testosterone (cFT) progressively decreased. Based on cFT evaluation, the proportion of hypogonadic patients progressively increased with a cumulative prevalence of 87.5% over the study course. A negative correlation was observed between serum mitotane levels >14 mg/L and TT and cFT.

**Conclusion:**

Testosterone deficiency is common in men with ACC prior to mitotane treatment. In addition, this therapy exposes these patients to further elevated risk of hypogonadism that should be promptly detected and counteracted, since it might have a negative impact on quality of life.

## Introduction

Mitotane, a derivative of the pesticide dichlorodiphenyltrichloroethane (DDT), is the only systemic therapy approved for the management of adrenocortical carcinoma (ACC) (1), an extremely rare disease with an estimated incidence in western countries ranging between 0.7 and 2 new cases per million population per year (2). The drug, either administered alone (2) or in combination with chemotherapy (3, 4), the EDP regimen (etoposide, doxorubicin, and cisplatin), still represents the only standard systemic therapy for ACC patients with locally advanced or metastatic disease who are not eligible for surgery (5, 6). Mainly thanks to the results of a retrospective study, which compared the outcome of ACC patients using or not using mitotane in adjuvant setting (7, 8), this therapy is recommended by the current international guidelines (5, 6) in patients undergoing radical surgery who are at high risk of relapse and death.

Mitotane is a drug difficult to manage, due to its long half-life, dose-limiting toxicity, and narrow therapeutic window. The strategy of mitotane administration is to achieve and maintain over time circulating drug concentrations ranging between 14 and 20 mg/L (9–11). Unfortunately, mitotane therapy leads to several adverse effects ranging from limited to severe, the most common toxicities involving the endocrine, gastrointestinal, and central nervous systems (12). The side effects are obviously increased when mitotane is administered in association with chemotherapy (13). As regard the endocrine side effects, mitotane therapy induces a deep inhibition of adrenal cortical function resulting in hypoadrenalism and the need in all patients for glucocorticoid supplementation, with individualized dosing, being tapered on the basis of a patient’s clinical symptoms and signs of hypoadrenalism (e.g. fatigue, muscle weakness) (14). Moreover, mitotane affects thyroid function, causing central hypothyroidism, burdened by asthenia and concentration deficits, which can ameliorate with the levothyroxine replacement therapy (12, 15).

Hypogonadism (low testosterone) in men is also frequently observed with a reported frequency ranging between 26% and 57% (12). This side effect is potentially relevant, since low testosterone has impacts on many organs (such as the cardiovascular, bones, adipose tissue, and skeletal muscles) and general well-being, and it may worsen asthenia, fatigue, and depression commonly reported by mitotane-treated patients. Although testosterone replacement therapy may ameliorate these symptoms (12), hypogonadism during mitotane treatment is underestimated and poorly characterized, and as a consequence, testosterone therapy is often started late or never considered (12, 13). This is an important issue particularly in ACC patients in whom the drug is administered in adjuvant setting, since they are young, disease free, and potentially cured, and receive the drug for a long period of time. Furthermore, the possible causes and onset timing of hypogonadism have not been well-defined in the literature. In fact, hypogonadism could result from primary deficiency of testosterone production, secondary deficiency from hypothalamus–pituitary dysfunction, or from increased production of sex hormone binding globulin (SHBG) from the liver that reduces the free amount of testosterone (representing the active form).

In this study, we retrospectively assessed the series of ACC male patients treated at the Medical Oncology of the Spedali Civili in Brescia, a reference center for this rare disease in Italy, with the aim of assessing the frequency of testosterone deficiency before and after mitotane therapy, the possible mechanisms involved, and the correlation between hypogonadism with serum mitotane levels and prognosis.

## Methods

This is a retrospective longitudinal study. Data of the patients from the Oncology Unit of Spedali Civili in Brescia, from January 2013 to September 2021, were collected, according to the following inclusion criteria: male sex, age ≥ 18 years, histological diagnosis of ACC, availability of data regarding gonadal and adrenal androgens biochemical profiles performed prior to mitotane treatment (alone or in association with chemotherapy) and at least once during therapy, blood samples at fasting in the morning between 8:00 and 10:00 analyzed at the central laboratory of University Hospital of Brescia (chemiluminescence immunoassay).

The primary endpoint of the study was the prevalence of male hypogonadism in men with ACC under adjuvant mitotane therapy. Secondary endpoints were the correlation between serum mitotane levels and gonadal function over time of therapy (up to 36 months), the possible mechanisms involved in the onset of hypogonadism, and the impact of testosterone deficiency on prognosis.

Anthropometric data, smoke and alcohol habits, ACC, and treatment information were collected for all the included patients. ACC hormone secretion was defined as elevated biochemical values of adrenal and gonadal hormones (cortisol, adrenal androgens, aldosterone, and estrogens). In detail, cortisol hypersecretion was defined as basal blood values of cortisol > 20 μg/dl and ACTH < 10 pg/ml (not in the course of glucocorticoids therapy). The presence of high-dose glucocorticoids therapy was defined as treatment with cortone acetate ≥50 mg daily or equivalent (due to clinical indication for the management of hypoadrenalism in course of adjuvant treatment).

At baseline (in detail, after adrenal surgery and prior to mitotane therapy) and after 3, 6, 12, 24, and 36 months of this adjuvant treatment, the following were analyzed: serum mitotane levels, total testosterone (TT), SHBG, calculated free testosterone (cFT) using Vermeulen formula (available at http://www.issam.ch/freetesto.htm), estradiol (E2), hemoglobin, hematocrit, creatinine, androstenedione, dehydroepiandrosterone sulfate (DHEAS), and 17-hydroxy progesterone (17OH-P).

To detect hypogonadism, testosterone deficiency was defined based on the biochemical finding of low TT and/or cFT. In details, cut-off values for defined low TT and cFT were <3.5 ng/ml and <63 pg/ml, respectively (16, 17). Eugonadal patients were defined for values of TT ≥3.5 ng/ml and cFT ≥63 pg/ml. In patients receiving testosterone therapy, its possible benefits were evaluated in terms of patient-reported clinical improvement in asthenia, muscle strength, and perceived well-being.

### Statistical analysis

Statistical Package for the Social Sciences software IBM SPSS Statistics, Version 25.0, Armonk, (NY) was used for statistical analysis. Since the variables were not normally distributed (Kolmogorov–Smirnov test was used), the comparison between quantitative variables was performed with Wilcoxon test, whereas the comparison between categorial variables was performed with chi-square test. During follow-up, the comparison between quantitative variables over time was performed with ANOVA test. Correlation between serum mitotane levels and biochemical data was performed with Spearman correlation. Kaplan–Meier curves were designed to assess the impact of testosterone deficiency on progression-free survival (PFS, defined as months without metastasis) and overall survival (OS). p-values<0.05 were considered statistically significant.

## Results

Of the 61 men evaluated for inclusion, 24 patients met eligibility inclusion criteria (**Supplementary Figure S1**). Data at baseline (after adrenal surgery and prior to the start of mitotane treatment) are shown in **Table 1**. At diagnosis, 10 patients (41.7%) were already hypogonadal (nine with low TT and one with low cFT). Of the latter, none had other known testicular–pituitary disease or a recent significant change in body weight or were taking high-dose glucocorticoid therapy or drugs interfering with gonadal function. No differences were observed regarding hypogonadism prevalence in men with secreting (cortisol and/or other adrenal–gonadal hormones) vs. non-secreting ACC and in patients with metastatic vs. non-metastatic tumor.

**Table 1:** Baseline characteristics of the included patients (n=24) prior to the start of mitotane.

Age at diagnosis (years)		52.5 (42.8-58)
BMI (kg/m^2^)		24.5 (22-25.8)
Smoking habit	Current smokers	3 (12.5%)
	Past smokers	9 (37.5%)
Alcohol habit	Regular drinkers	7 (29.2%)
	Occasional drinkers	5 (20.8%)
ACC side (left)		15 (62.5%)
ACC secretion	Non-secreting ACC	14 (58.3%)
	Only cortisol	5 (20.8%)
	Cortisol + adrenal androgens	2 (8.3%)
	Cortisol + aldosterone	1 (4.2%)
	Cortisol + estrogens	1 (4.2%)
	Only estrogens	1 (4.2%)
Metastatic ACC		15 (62.5%)
Hypogonadism		10 (41.7%)

Data are expressed as median (IQR) for continuous variables and as absolute number (%) for categorical variables. IQR, interquartile range; BMI, Body Mass Index; ACC, adrenocortical carcinoma.

The comparison between hypogonadal and eugonadal patients at baseline (prior to adjuvant treatment) is shown in **Table 2**. No significant differences were found in terms of age at diagnosis of ACC, Body Mass Index (BMI), smoke or alcohol habits, and ACC characteristics. At baseline, apart from TT levels, only DHEAS was significantly different between patients with hypogonadism and eugonadism (3.7 vs. 0.3 µg/ml; p=0.001); the two patients affected by secreting adrenal androgens ACC with high DHEAS levels (>6 µg/ml) were present in the hypogonadal group.

**Table 2:** Comparison between hypogonadal and eugonadal patients prior to the start of mitotane.

		Hypogonadal patients (N=10)	Eugonadal patients (N=14)	p
BMI (kg/m^2^)		25.0 (22.5-25.0)	23.0 (21.0-27.0)	0.787
Age at diagnosis (years)		49.0 (41.5-57.0)	54.5 (41.8-64.0)	0.463
Smoking habit	Current smokers	3 (30.0%)	0 (0.0%)	0.061
	Past smokers	2 (20.0%)	7 (50.0%)	0.061
Alcohol habit	Regular drinkers	2 (20.0%)	5 (35.7%)	0.243
	Occasional drinkers	1 (10.0%)	4 (28.6%)	0.243
Secreting ACC		5 (50.0%)	5 (35.7%)	0.423
Metastatic ACC		6 (60.0%)	9 (64.3%)	0.825
Haemoglobin (g/dl)		13.5 (11.7-15.4)	13.6 (12.5-14.3)	0.777
Haematocrit (%)		41.1 (36.0-45.3)	39.4 (37.6-42.7)	0.549
Creatinine (mg/dl)		0.7 (0.5-0.9)	0.8 (0.7-1.1)	0.139
TT (ng/ml)		2.4 (1.4-3.1)	5.5 (4.0-6.8)	**0.001**
SHBG (nmol/l)		53.0 (24.8-65.5)	59.5 (30.3-71.5)	0.663
cFT (pg/ml)		65.3 (59.2-98.1)	97.4 (69.6-121.4)	0.125
E2 (pg/ml)		25.0 (18.0-26.0)	23.5 (12.8-32.0)	0.764
Androstenedione (ng/ml)		1.7 (0.9-3.6)	0.8 (0.6-1.1)	0.065
DHEAS (µg/ml)		3.7 (1.4-6.5)	0.3 (0.2-0.7)	**0.001**
17OH-P (ng/ml)		3.4 (1.1-3.7)	1.4 (1.2-1.7)	0.073

Data are expressed as median (IQR) for continuous variables and as absolute number (%) for categorical variables. A comparison between continuous variables was performed with Wilcoxon test; a comparison between categorical variables was performed with chi-square test. IQR, interquartile range; BMI, Body Mass Index; ACC, adrenocortical carcinoma; TT, total testosterone; SHBG, sex hormone binding globulin; cFT, calculated free testosterone; E2, estradiol; DHEAS, dehydroepiandrosterone; 17OH-P, 17-hydroxyprogesterone; significant data in bold.

Median age at start of mitotane therapy was 52.5 years (43.5–58.8), and median duration of treatment was 23.5 months (11.75–33.75). The median time to attain serum mitotane levels within the therapeutic range (defined as mitotanemia > 14 mg/l) was 4 months (3.5–10.5). In 14 cases (58.3%), mitotane was given alone, whereas in 10 patients (41.6%), it was combined with chemotherapy.

**Table 3** shows the variation in biochemical and clinical parameters during treatment with mitotane. TT showed a biphasic evolution with the progressive increase in serum mitotane levels over time: it significantly increased in the first 6 months and then decreased progressively until 36 months. On the contrary, SHBG progressively and significantly increased over the entire follow-up period, and as a consequence, cFT progressively and significantly decreased over time. Therefore, the prevalence of hypogonadism during the follow-up was unchanged when considering TT but significantly increased when considering cFT. Besides the 10 patients with testosterone deficiency before mitotane therapy (considering TT and/or cFT), 11/14 men (78.6%) developed hypogonadism during the treatment, with seven of them showing low cFT with normal TT levels. The cumulative prevalence of testosterone deficiency over the course of the study was therefore 87.5% (21/24 patients).

**Table 3:** Variation in biochemical and clinical parameters and serum mitotane levels during follow-up.

	Before mitotane (n=24)	3 months (n=21)	6 months (n=22)	12 months (n=17)	24 months (n=14)	36 months (n=10)	p
Haemoglobin (g/dl)	13.5 (12.5-14.8)	13.0 (11.1-13.5)	12.2 (11.1-12.9)	12.4 (11.8-13.8)	13.9 (12.8-14.6)	13.9 (12.9-14.4)	**0.041**
Haematocrit (%)	40.2 (37.7-43.2)	38.5 (33.2-40.6)	36.5 (33.4-37.9)	38.6 (35.6-40.9)	40.9 (36.9-42.7)	41.1 (38.4-42.2)	**0.048**
Creatinine (mg/dl)	0.8 (0.7-1.1)	0.8 (0.5-1.0)	0.7 (0.6-0.8)	0.9 (0.8-1.3)	1.1 (0.8-1.3)	0.8 (0.7-1.0)	0.106
BMI (kg/m^2^)	23.6 (22.2-25.9)	24.5 (22.3-25.9)	25.4 (22.4-26.1)	23.8 (21.9-25.2)	23.2 (22.7-26.0)	23.9 (22.4-26.3)	0.322
TT (ng/ml)	3.8 (2.5-5.9)	7.7 (3.9-11.6)	7.8 (3.8-11.7)	4.7 (2.7-8.8)	4.3 (2.9-6.1)	3.6 (2.6-7.4)	**0.043**
SHBG (nmol/l)	55.0 (22.0-66.0)	126.0 (65.5-213.5)	152.5 (78.0-192.5)	157.5 (98.8-234.8)	195.0 (158.8-406.8)	194.0 (126.0-402.0)	**0.010**
cFT (pg/ml)	65.3 (57.2-78.2)	53.5 (23.5-109.9)	47.5 (23.5-87.0)	36.6 (11.5-67.3)	18.3 (5.9-28.5)	17.7 (9.2-25.3)	**0.017**
TT < 3.5 ng/ml	9/24 (37.5%)	4/21 (19.0%)	5/22 (22.7%)	6/17 (35.3%)	5/14 (35.7%)	4/10 (40.0%)	0.538
cFT < 63 pg/ml	1/6 (16.7%)	9/15 (60.0%)	10/14 (71.4%)	9/11 (81.8%)	8/9 (88.9%)	6/6 (100.0%)	**0.037**
E2 (pg/ml)	24.0 (15.5-29.0)	25.0 (14.0-32.8)	27.0 (19.0-39.5)	32.0 (20.5-45.0)	25.0 (16.0-26.0)	25.0 (19.6-29.0)	0.402
Androstenedione (ng/ml)	0.9 (0.8-2.3)	1.2 (0.4-2.7)	0.9 (0.4-2.1)	0.4 (0.3-0.9)	0.3 (0.2-0.7)	0.6 (0.2-0.9)	**0.048**
DHEAS (µg/ml)	0.5 (0.2-3.5)	0.2 (0.2-0.6)	0.2 (0.2-0.5)	0.2 (0.2-1.5)	0.2 (0.2-0.5)	0.2 (0.1-2.1)	0.110
17OH-P (ng/ml)	1.5 (1.2-1.9)	1.9 (1.5-2.6)	2.0 (1.4-3.0)	1.5 (0.9-2.0)	1.2 (0.6-1.6)	1.2 (0.4-1.9)	0.093
Mitotanemia (mg/l)	–	8.0 (3.8-11.2)	9.5 (6.3-13.9)	13.2 (10.8-17.2)	14.4 (11.0-16.5)	14.3 (10.0-19.0)	**0.018**

Data are expressed as median (IQR) for continuous variables and as absolute number (%) for categorical variables. Comparison between continuous variables was performed with ANOVA test for repeated measures (including post-hoc Tukey’s analysis); a comparison between categorical variables was performed with chi-square test. IQR, interquartile range; TT, total testosterone; SHBG, sex hormone binding globulin; cFT, calculated free testosterone; E2, estradiol; DHEAS, dehydroepiandrosterone; 17OH-P, 17-hydroxy progesterone; BMI, Body Mass Index; significant data in bold.

During mitotane treatment, 91.7% (22/24) of patients required high-dose glucocorticoid therapy. There were no significant differences in hypogonadism prevalence with respect to the presence or absence of high-dose glucocorticoid therapy (20/22 vs. 1/2, p=0.094). At all times of evaluation, there was no significant difference in testosterone deficiency prevalence between men treated with mitotane alone with respect to those with also chemotherapy.

**Figure 1:**
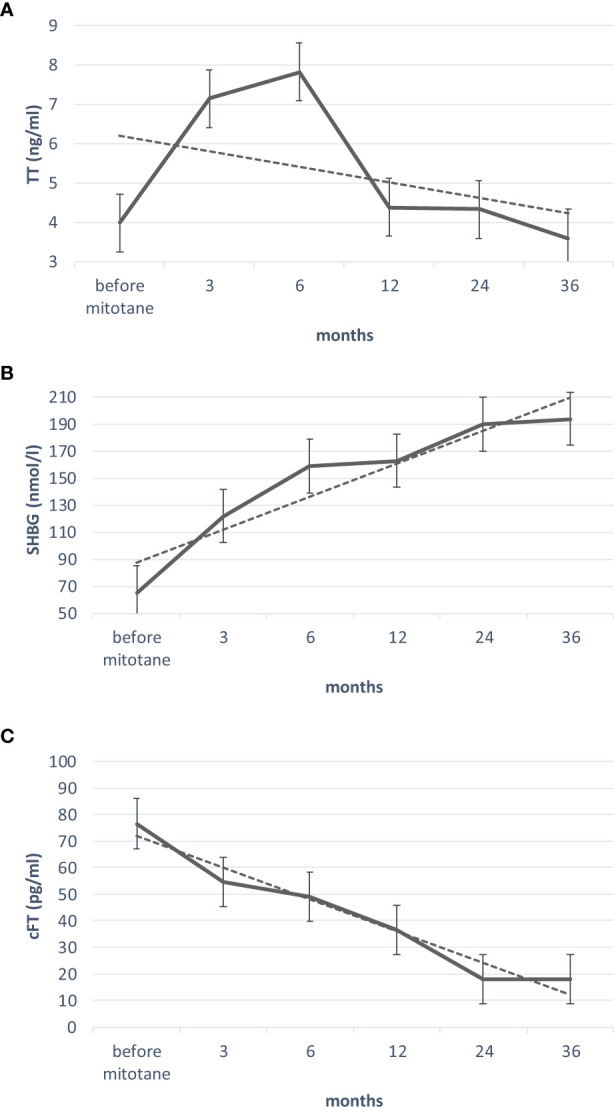
Trend of TT **(A)**, SHBG **(B)** and cFT **(C)** levels, during mitotane treatment. Friedman test was used to develop the trend lines. TT, total testosterone; SHBG, sex hormone binding globulin; cFT, calculated free testosterone. Trend lines in dotted.

**Figure 1** shows the median trend of TT, SHBG, and cFT levels during mitotane treatment. A significant reduction in hemoglobin and hematocrit was found in the first 6 months of treatment. Androstenedione increased in the first 3 months and then progressively decreased, whereas a progressive decrease in DHEAS was observed without statistical significance.

The correlation between serum mitotane levels over time, and gonadal axis variations is shown in **Table 4**. Within the first 6 months of mitotane treatment, a positive correlation was shown between serum mitotane levels and testosterone deficiency and between serum mitotane levels and SHBG levels, especially when mitotane levels were >14 mg/L. Similarly, a negative correlation was observed between serum mitotane levels >14 mg/L and TT and cFT. The positive correlation between mitotane levels and testosterone deficiency was confirmed at 36 months. No correlations were found between mitotane levels and the other hormones, and between BMI with respect to mitotane levels and gonadal axis (TT, SHBG, and cFT).

**Table 4:** Correlations between serum mitotane levels and gonadal function over time.

Serum mitotane levels	3 mo (mg/L)	3 mo > 14 mg/L	6 mo (mg/L)	6 mo > 14 mg/L	12 mo (mg/L)	12 mo. > 14 mg/L	24 mo (mg/L)	24 mo. > 14 mg/L	36 mo (mg/L)	36 mo. > 14 mg/L
TT (ng/ml)	0.298	-0.401	-0.271	**-0.446** *	-0.231	-0.364	0.093	0.301	-0.351	-0.218
SHBG (nmol/l)	0.342	**0.586** *	0.420	**0.626** *	-0.086	0.126	0.429	0.504	0.714	0.577
cFT (pg/ml)	-0.414	-0.537	-0.441	**-0.713** **	-0.200	-0.252	-0.214	-0.126	0.000	-0.707
Hypogonadism	**0,505** *	**0.632** **	0.276	**0.486** *	0.186	0.260	-0.215	0.183	**0.635** *	**0.688** **

Correlation between variables was performed with Spearman correlation. TT, total testosterone; SHBG, sex hormone binding globulin; cFT, calculated free testosterone; mo, months. Number of patients: 3 mo=21; 6 mo=22; 12 mo=17; 24 mo=14; 36 mo=10.

*Correlation is significant at p=0.05 (two-tail).

** Correlation is significant at p=0.01 (two-tail).

significant data in bold.

**Figure 2:**
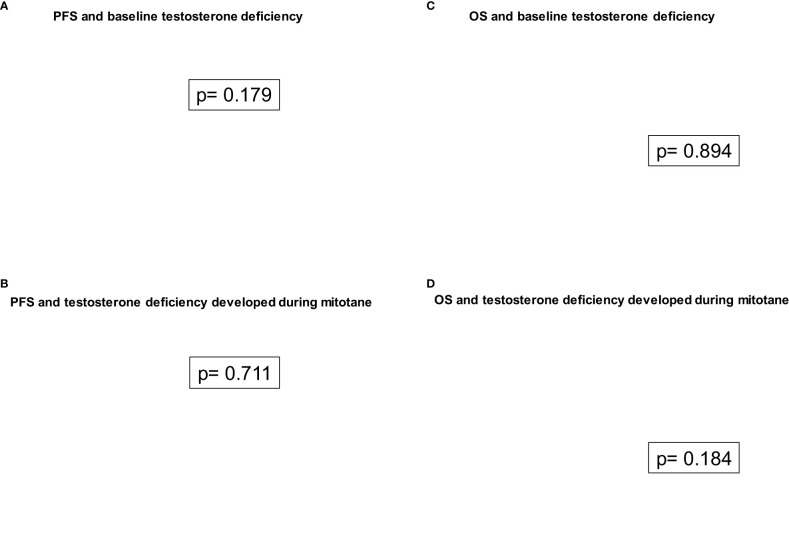
Impact of testosterone deficiency on PFS and OS. **(A)** Baseline testosterone deficiency and PFS: cases of metastasis in men with baseline testosterone deficiency (10/10) vs. baseline eugonadal men (11/14); **(B)** testosterone deficiency developed during treatment and PFS: cases of metastasis in men with developed testosterone deficiency (9/11) vs. eugonadal men also during treatment (2/3); **(C)** baseline testosterone deficiency and OS: cases of death in men with baseline testosterone deficiency (3/10) vs. baseline eugonadal men (5/14); **(D)** testosterone deficiency developed during treatment and OS: cases of death in men with developed testosterone deficiency (5/11) vs. eugonadal men also during treatment (0/3). Comparison was performed with chi-square test. Continuous line: testosterone deficiency patients; dotted line: eugonadal men. On the horizontal axis, the months passed. PFS, progression-free survival (as months without metastasis); OS, overall survival (as months without death).

By the end of the whole study period (36 months), 21 patients (87.5%) had metastatic ACC and eight (33.3%) died, of which three were hypogonadal prior to the start of mitotane treatment and five had developed testosterone deficiency during follow-up. However, testosterone deficiency (both at baseline and developed during the treatment) had no impact on PFS and OS (**Figure 2**).

During the study (after 12 or 24 months of mitotane treatment), four men received testosterone replacement therapy (in these four patients, the subsequent serum gonadal hormonal measurements—modified by testosterone therapy—were not considered to calculate the prevalence of hypogonadism). Of them, two were treated with injective undecanoate testosterone 1 g every 3 months and two with transdermal testosterone 20 mg daily. Substitution therapy was started based on the presence of sexual symptoms, severe asthenia, and reduced muscle strength. A slight clinical improvement was reported by these patients after 6 months of testosterone treatment.

## Discussion

In this single-center retrospective study involving male ACC patients undergoing treatment with mitotane, we showed a relevant proportion of testosterone deficiency with a cumulative prevalence over the course of the study of 87.5%. These data expand the results of previous studies (12, 15, 18, 19); nevertheless, we found a higher proportion of hypogonadal patients (reported from 26% to 57% in the previous studies) (12). This difference could be related to two main factors: the longer follow-up period of our study and the more detailed analysis of hypogonadism based on both TT and cFT.

Noteworthy, 40% of our patients reported testosterone deficiency already before starting mitotane treatment (after adrenal surgery), and the prevalence further increased during the 36 months of follow-up. The proportion of testosterone deficiency in our series did not change in relation to the presence or absence of metastatic disease and the ACC secretory status. These data suggest that neither the tumoral extension nor the tumoral hormonal hypersecretion influenced this phenomenon. Instead, the high prevalence of testosterone deficiency at baseline could be explained by two factors. First of all is the fragility of these patients, given the tumoral condition, with a consequent detrimental effect on gonadal function and reduced production of testosterone. In addition, due to ACC, a possible subversion of adrenal steroidogenesis, with consequent reduction in the amount of testosterone produced by the adrenal glands, cannot be excluded. In particular, an arrest in the last stages of adrenal steroidogenesis (above all due to enzymatic deficiency of 17-beta hydroxysteroid dehydrogenase) can result in the accumulation of androgen precursors (DHEAS, 17-OH progesterone, and androstenedione), reducing the conversion to testosterone (20). Indeed, serum levels of androgen precursors (above all DHEAS) were higher in men with testosterone deficiency than in eugonadal patients at baseline conditions (in part, probably also because of the two patients with secreting adrenal androgens ACC were in the hypogonadal group). This observation confirms the importance of evaluating the androgenic profile also in the male ACC patients, as recommended by international guidelines (5, 6).

On the other hand, the most important factor contributing to the development of testosterone deficiency during the treatment with mitotane is the progressive increase in SHBG levels, produced by the liver through an estrogenic-like effect of mitotane already described (mediated by the induction of transcription factors for SHBG) (12, 15, 21). The increased amount of SHBG binds more testosterone resulting in an increase in TT levels and a decrease in free testosterone (which is the active form of the hormone), with a consequent alteration of testosterone production (22). In fact, during mitotane treatment, most patients were diagnosed with hypogonadism due to low cFT values, with normal, or even elevated, TT levels. Therefore, our results strongly suggest that cFT assessment is essential for the evaluation of the male gonadal status, as also recommended by recent guidelines (17, 23) in the general population. In addition, despite a not significant difference (given the small number of cases), another mechanism that contributes to the development of hypogonadism in these patients is the high-dose glucocorticoid therapy (due to its known interfering effect on male gonadal axis) (15). The latter is necessary in most patients during adjuvant treatment, given the increase in cortisol binding globulin (CBG) induced by mitotane (with a consequent need of further increased therapy) (15).

In details, our data revealed a biphasic change in TT levels with a significant increase in the first 6 months and then a progressive decrease. This initial increase in testosterone levels was also observed in a previous paper by Basile and colleagues (15). However, in that study, the increase in TT levels was maintained throughout an observation period of 24 months, and this observation is in contrast with our data. We do not have an explanation for this discrepancy. More interestingly is the evolution of cFT levels that progressively reduced over time, due to the observed strong increase in SHBG values. From a pathophysiological point of view, our hypothesis is that the reduction in free testosterone induces a pituitary compensation similarly to what has been described in other populations with elevated SHBG, as men with human immunodeficiency virus under retroviral therapy (24). In particular, as SHBG increases, the pituitary production of luteinizing hormone (LH) increases due to lower negative feedback by free testosterone, and therefore, Leydig cells are stimulated to produce higher amount of testosterone to gain a new steady state, as suggested by de Ronde et al. (22). Once this compensation mechanism is exhausted, TT also falls (22, 24). This possible mechanism to explain the observed biphasic TT trend in men treated with mitotane is suggested also by the finding of Basile et al. who observed an increase in LH levels in the first months of treatment (15). Unfortunately, LH was not available in our patients to support these data or, otherwise, to observe an eventual depressing effect of mitotane on LH levels (with consequent possible further drop in TT values).

Interestingly, we observed an inverse correlation between mitotane levels and testosterone concentrations (and a direct relationship with SHBG serum levels) mainly in patients who achieved mitotane concentrations above the lower limit of the therapeutic range (14 mg/L). This underlines that the achievement of therapeutic values is crucial to make the mitotane fully biologically active, but this raises the risk of development of hypogonadism.

The frequency of testosterone replacement therapy is relatively low (four patients) in spite of the high prevalence of testosterone deficiency. This is due to the fact that, according to guidelines, we have addressed in testosterone therapy only patients who spontaneously reported typical symptoms of low testosterone (such as sexual dysfunction) and/or aspecific symptoms as asthenia and reduced muscle strength. However, given the complexity of these patients in whom these symptoms might also be related to the general conditions, it is possible that they under-considered or not reported these symptoms. In light of the results of our study, we stress the need for a more thoughtful andrological evaluation. Although we did not observe a relationship between low testosterone and overall/progression free survival (perhaps also due to the small sample size), we cannot exclude the fact that tumoral prognosis could impair testosterone levels and/or testosterone deficiency might further worsen the outcome of these patients. Therefore, testosterone replacement therapy should be carefully evaluated and proposed in male patients with low testosterone given mitotane therapy. Such therapy could have an impact not only on the quality of life and perceived well-being but also on general symptoms, such as asthenia, muscle strength, bone health, and anemia (23). In addition, testosterone therapy might represent a support care against cancer-related fatigue, inappetence, cachexia, and depression (25).

The strengths of this study rely on the strict selection criteria of a consecutive series of ACC male patients undergoing mitotane therapy and followed for a long period in a single center. Furthermore, the hormone assessment was performed in the same laboratory and included the complete panel of testicular and adrenal steroids. Furthermore, this is the first study focused on the andrological assessment of patients treated with mitotane with hypogonadism as primary endpoint. The retrospective nature and absence of LH determination (the latter is fundamental for hypogonadism classification) are the main limitations; the use of immunoassay instruments (instead of mass spectrometry) to detect testosterone levels is another limiting point.

In conclusion, testosterone deficiency is common in men with ACC before and in the course of mitotane treatment, and this therapy exposes these patients to an elevated risk of hypogonadism, which might negatively impact the quality of life. This is a relevant issue in this patient setting, since ACC often occurs when patients are at the high of their working and social life, which are significantly affected by complex feelings and lifestyle changes due to the cancer diagnosis.

Based on the results of this study, the evaluation of testosterone deficiency (hypogonadism) should be systematically performed in male ACC patients, and the determination of free testosterone is mandatory in men treated with mitotane. Most of these patients require an andrological evaluation to assess the feasibility and potential utility of testosterone replacement therapy. Collaboration between medical oncologists and andrologists is therefore necessary for the management of these complex patients.

## Data availability statement

The raw data supporting the conclusions of this article will be made available by the authors, without undue reservation.

## Ethics statement

The studies involving human participants were reviewed and approved by Ethics committee ASST Spedali Civili of Brescia. The patients/participants provided their written informed consent to participate in this study.

## Author contributions

AD and DC took the lead in writing the manuscript. AB, AF, and CC conceived the design of the study. AD, DC, PF, ML, LCP, VC, and AA collected and provided the clinical data. SG, AB, AF, and CC supervised the project and contributed to the interpretation of the clinical data. All authors actively revised the manuscript provided critical feedback. All authors contributed to the article and approved the submitted version.
