# Supplementary material for: Androgen serum levels in male patients with adrenocortical carcinoma given mitotane therapy: A single center retrospective longitudinal study
Source: Front Endocrinol (Lausanne). 2023 Apr 5;14:1128061. doi: 10.3389/fendo.2023.1128061 (PMC10108714; doi:10.3389/fendo.2023.1128061)

Supplementary Meterial

Figure 1. Consort diagram on inclusion criteria. ACC= adrenocortical carcinoma.

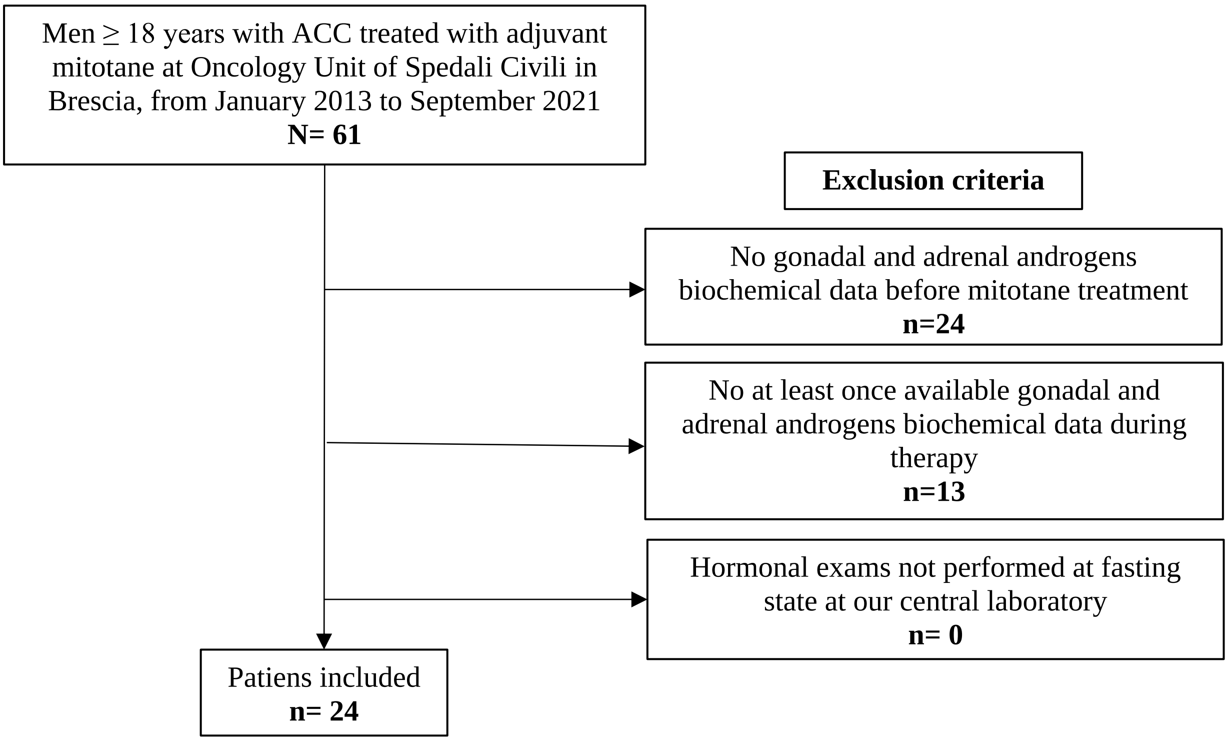

Supplement: Supplementary file 1 [file Image_1.pdf]
